# Eculizumab Therapy Leads to Rapid Resolution of Thrombocytopenia in Atypical Hemolytic Uremic Syndrome

**DOI:** 10.1155/2014/295323

**Published:** 2014-10-22

**Authors:** Han-Mou Tsai, Elizabeth Kuo

**Affiliations:** ^1^iMAH Hematology Associates, New Hyde Park, NY 11040, USA; ^2^Department of Medicine, University of Texas Southwestern School of Medicine, Dallas, TX 75235, USA

## Abstract

Eculizumab is highly effective in controlling complement activation in patients with the atypical hemolytic uremic syndrome (aHUS). However, the course of responses to the treatment is not well understood. We reviewed the responses to eculizumab therapy for aHUS. The results show that, in patients with aHUS, eculizumab therapy, when not accompanied with concurrent plasma exchange therapy, led to steady increase in the platelet count and improvement in extra-renal complications within 3 days. By day 7, the platelet count was normal in 15 of 17 cases. The resolution of hemolytic anemia and improvement in renal function were less predictable and were not apparent for weeks to months in two patients. The swift response in the platelet counts was only observed in one of five cases who received concurrent plasma exchange therapy and was not observed in a case of TMA due to gemcitabine/carboplatin. In summary, eculizumab leads to rapid increase in the platelet counts and resolution of extrarenal symptoms in patients with aHUS. Concurrent plasma exchange greatly impedes the response of aHUS to eculizumab therapy. Eculizumab is ineffective for gemcitabine/carboplatin associated TMA.

## 1. Introduction

Many patients with the diagnosis of atypical hemolytic uremic syndrome (aHUS) are found to have defective regulation of the alternative complement system [1]. This discovery leads to the use of a humanized monoclonal antibody of complement C5, eculizumab, for aHUS [2]. However, presently available laboratory tests do not identify all patients with defective complement regulation. The tests also have very lengthy turnaround times. Consequently, clinicians have to initiate eculizumab therapy based on a presumptive diagnosis of aHUS. A positive response to eculizumab therapy helps confirm the diagnosis of aHUS. However, there is little data to determine how long the treatment should be continued before a patient's disorder is considered unresponsive to eculizumab therapy.

Mechanistically the complications of aHUS belong to four groups: renal dysfunction and hypertension due to ischemic or membrane attack complex- (MAC-) mediated glomerular injury; thrombocytopenia due to platelet consumption in thrombosis; microangiopathic hemolytic anemia (MAHA) due to abnormal shear stress created by thrombosis or intimal swelling; and extrarenal complications such as edema of the brain, lung, or the digestive organs due to abnormal vascular permeability mediated by anaphylatoxins C3a and C5a [3].

We hypothesize that thrombocytopenia and extrarenal symptoms should begin to resolve soon after complement activation is suppressed by eculizumab, when no new thrombosis occurs and no additional anaphylatoxins are released. In contrast, improvement in renal function depends on the reversibility of glomerular injury and the resolution of arteriolar stenosis. In TMA, arteriolar stenosis results from a variable mix of endothelial swelling, which is expected to resolve quickly when complement activation ceases; and thrombosis, which is dissolved by the fibrinolytic system at slower paces. Consequently, improvement in the renal function in response to anticomplement therapy is likely to be quite variable. Similarly, remission of MAHA, also depending on the resolution of arteriolar stenosis, is expected to be variable. In patients with arteriolar stenosis predominated by endothelial swelling, MAHA should resolve quickly. On the other hand, in patients with extensive arteriolar thrombosis or subendothelial fibrosis, resolution of MAHA may take weeks to months. To explore the merits of this hypothesis, we have reviewed the records of patients who were treated with eculizumab for aHUS.

## 2. Methods

The records of patients who were treated with eculizumab for presumptive diagnosis of aHUS were reviewed. Patients presenting with MAHA, thrombocytopenia, and renal failure were considered to have aHUS if the plasma ADAMTS13 activity was greater than 10% and other causes of the syndrome of MAHA and thrombocytopenia such as DIC, systemic autoimmune disorders, lupus anticoagulants, metastatic neoplasms, Shiga toxin, or neuraminidase associated HUS were excluded. Molecular analysis was performed (Molecular Otolaryngology and Renal Research Laboratories, University of Iowa) to detect mutations of* complement factor H* (*CFH*),* membrane cofactor protein* (*MCP*),* complement factor I*,* complement factor B*,* C3*, and* thrombomodulin*; copy numbers of* complement factor H-related protein 1* (*CFHR1*); and antibodies of CFH. Plasma ADAMTS13 activity levels were performed (the Blood Center of Wisconsin, Milwaukee, WI) to exclude the diagnosis of TTP. The diagnosis of aHUS was considered confirmed if one or more genetic alterations or CFH antibodies affecting the regulation of the alternative complement pathway were detected or the patient showed a clear response to eculizumab therapy.

We also reviewed case records in the literature via search of PubMed using the terms of atypical hemolytic uremic syndrome and eculizumab and included cases with platelet counts within the first 7 days for assessment.

## 3. Results

During a period of three years, the authors encountered 7 cases of presumed or confirmed aHUS, of which 5 were treated with eculizumab and were further analyzed for this report. For comparison, 10 cases of acquired TTP, 2 cases of hereditary TTP, and one case of MAHA and thrombocytopenia presumably due to lupus vasculitis which had resolved by the time of evaluation were encountered during the same period.

The demographics, comorbid conditions, presenting features, pathological findings of kidney biopsy, and results of molecular testing are summarized in Table 1. Case 1 presented with bloody diarrhea and was initially suspected to have Shiga toxin associated HUS. However, her stools were negative for Shiga toxins. Case 2, previously reported [4], presented three months after undergoing autologous hematopoietic stem cell therapy (HSCT) for advanced multiple myeloma. Case 3, also previously reported [4], had recurrent episodes of severe hypertension accompanied with mild renal insufficiency and MAHA for four and a half years before he was found to have aHUS and treated with eculizumab. Case 4 presented at 22 weeks of her third pregnancy and was initially assumed to have preeclampsia; however, her disease persisted after termination of pregnancy. This patient was retreated for relapse 3 months after eculizumab therapy was discontinued. Case 5 had kidney biopsy performed for renal failure at 3 weeks after her seventh triweekly course of chemotherapy with gemcitabine and carboplatin for metastatic cholangiocarcinoma.

Four patients (Cases 1–4) presented with abdominal symptoms of pain, nausea, vomiting, and/or diarrhea. All five patients had altered mental status (confusion or somnolence). One had visual scotoma. Two patients had severe albeit brittle hypertension as high as 220/146 and 221/159 mmHg, respectively.

Kidney biopsy, performed in 3 patients (Cases 3–5), revealed the changes of thrombotic microangiopathy (TMA). Molecular testing detected complement factor H antibodies in the case after autologous HSCT (Case 2). This patient also had heterozygous genomic deletion of CFHR3 and CFHR1 and an uncommon nucleic acid sequence alteration (c.1697A>C, p.Glu566Ala) of complement factor B of undetermined significance. A rare alteration (c.3607C>T [rs145347741], p.Arg1203Trp) of CFH was detected in the case with onset during pregnancy (Case 4).

As expected, the laboratory tests revealed anemia with elevated LDH and thrombocytopenia (Table 2). In none of the patients was the platelet count less 50 × 10^9^/L. The serum creatinine concentration was 2.51−5.52 mg/dL at presentation, rising to higher levels in each case after admission. Dialysis was required in Cases 2 and 5.

The plasma ADAMTS13 activity was normal or slightly decreased in all cases. Four patients were treated with 5–17 sessions of plasma exchange before the treatment was switched to eculizumab. With plasma exchange, the platelet count increased in 3 patients and LDH decreased in all five patients. However, in none of the patients was the platelet count normalized or altered mental status or visual symptoms completely resolved (Table 3).

The responses to eculizumab therapy, also summarized in Table 3, are notable for the normalization of the platelet count by day 7 in each of the 5 courses of eculizumab therapy in Cases 1–4. The platelet count did not increase in the case of gemcitabine/carboplatin associated TMA. In that case, the platelet count was never normalized after 4 doses of eculizumab.

The responses in serum creatinine and LDH were variable, with none normalizing by day 7. Improvement of renal function by one stage was observed by day 14 during both courses of treatment in Case 4; by day 63 in Case 3; and by day 378 in Case 2. In Case 2, further improvement by one stage occurred by day 483. The LDH normalized by day 14 in Cases 3 and 4 and by day 70 in Case 2; it did not normalize by day 21 in Case 3 and by day 84 in Case 5.

**Figure 1:**
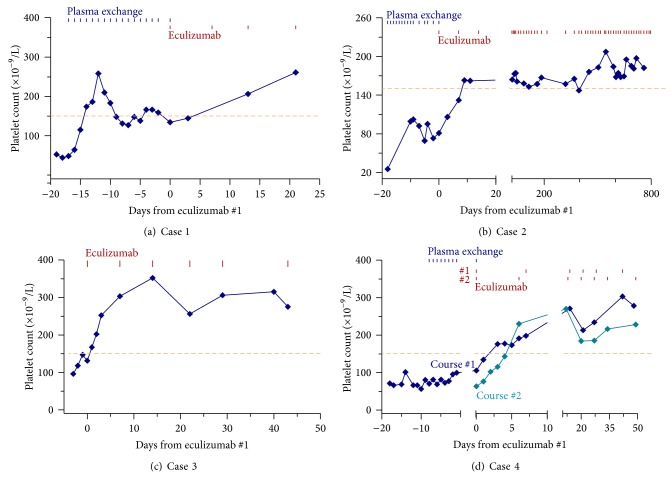
Rapid resolution of thrombocytopenia with eculizumab therapy for aHUS. In Cases 1–4 of aHUS (panels a–d), the platelet counts began to steadily increase within 3 days, normalizing by day 7 following initiation of eculizumab therapy. Such rapid and steady increase in the platelet count did not occur in three cases when they were treated with plasma exchange. The dashed lines mark the lower limit of normal platelet counts.

**Figure 2:**
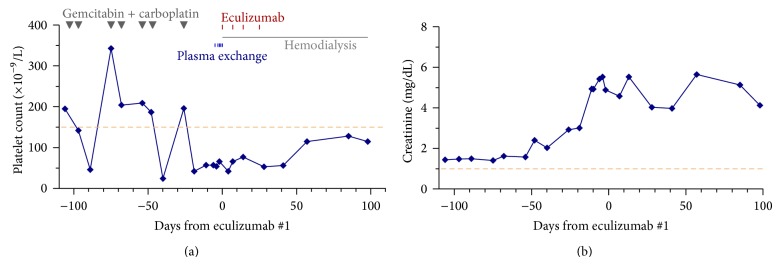
Development of thrombocytopenia and renal failure in a patient of metastatic cholangiocarcinoma treated with gemcitabine and carboplatin and the lack of response to eculizumab therapy. Neither plasma exchange nor eculizumab therapy increased the platelet counts or improved the renal function of this case. The thrombocytopenia induced by chemotherapy was transient before renal failure occurred. Thus, the lack of platelet response to eculizumab therapy was not due to bone marrow suppression by chemotherapy. The dashed lines mark the lower limit of normal platelet count and the upper limit of serum creatinine, respectively.

The courses depicted in Figure 1 show that the platelet count began to exhibit steady increase within 3 days in each of the 5 courses in Cases 1–4. No increase in the platelet count occurred in Case 5 while she was being treated with eculizumab (Figure 2).

From the literature, we identified 15 and 17 cases, respectively, in 9 reports with data available for analysis of the platelet count responses and its normalization by day 7 after the first dose of eculizumab therapy for aHUS (Table 4) [5–13]. Among the patients who were not receiving concurrent plasma exchange therapy, the platelet count began to show steady increase within 3 days in each of the 10 cases and was normalized by day 7 in 10 of 12 cases. In contrast, among the 5 patients with concurrent plasma exchange, only one showed immediate and steady increase in the platelet count, and none had normalized platelet counts by day 7. The difference in the platelet count response between the groups with and without concurrent plasma exchange was highly significant (*P* ≤ 0.001 by Fisher's exact test).

## 4. Discussion 

For patients presenting with the syndrome of MAHA, thrombocytopenia, and renal failure but no comorbid conditions, aHUS is the likely diagnosis once the diagnosis of TTP is excluded. Other causes of idiopathic TMA such as mutations of* diacylglycerol kinase epsilon* (DGKE) and cobalamin C disease are very rare or have not been described in adults [4, 14–16]. An association between aHUS and plasminogen mutations has been suggested but remains to be confirmed [17].

The diagnosis of aHUS is much less straightforward for patients with comorbid conditions such as HSCT, autoimmune disorders, pregnancy, or various drugs. In such cases, even the finding of TMA in kidney biopsy does not necessarily constitute the diagnosis of aHUS, because TMA may result via other mechanisms of endothelial injury [3]. In addition to Shiga toxins, microbial neuraminidases, and drugs such as bevacizumab that disrupts the vascular endothelial growth factor (VEGF) signaling pathway, TMA may occur in association with drugs such as gemcitabine and other chemotherapeutic agents via as yet known mechanisms.

For patients with a suspected diagnosis of aHUS, a trial of anticomplement therapy is warranted to prove or refute a role of defective complement regulation. Nevertheless, clinicians are often reluctant to institute the therapy because eculizumab is costly and associated with a small but definite risk of fulminant meningococcal infection. Once the therapy is started, clinicians are often confronted with the question of how to assess the response of aHUS to eculizumab. Should one expect simultaneous improvement in all the complications of aHUS, including thrombocytopenia, MAHA, renal failure, hypertension, and extrarenal complications? When is it safe to discontinue the therapy for lack of response?

In this study, we find eculizumab therapy leads to rapid resolution of thrombocytopenia and extrarenal symptoms of aHUS. Evidence of improvement in both is obvious by day 7, before the second dose of eculizumab. Lack of rapid resolution in thrombocytopenia or extrarenal complications may be taken as evidence against continuing eculizumab therapy beyond the first dose. In this regard, the lack of response in Case 5 after one dose of eculizumab suggests that gemcitabine/carboplatin associated TMA is not due to uncontrolled complement activation.

Our review of the literature identifies 12 additional cases with platelet count responses within 7 days of treatment that support our findings. Importantly, it also shows that concurrent plasma exchange greatly impedes the response of aHUS to eculizumab therapy. Since plasma exchange removes the infused medication, the delay in response is not unexpected. Historically, aHUS has been treated like TTP with plasma exchange. The response of aHUS to plasma exchange is variable and unpredictable. In 3 of our cases of aHUS, plasma exchange therapy resulted in partial improvement. However, the platelet counts did not normalize and the extrarenal symptoms persisted. Clinical trials have demonstrated that anticomplement therapy with eculizumab is superior to plasma exchange for the treatment of aHUS [2]. The responses in our cases of aHUS are consistent with this finding. Therefore, plasma exchange is now only used for the treatment of aHUS when eculizumab is not available or TTP is not yet excluded. For patients with aHUS or presumed aHUS, TTP should have been excluded and there is no reason to continue plasma exchange therapy when eculizumab therapy is initiated.

The resolution of MAHA and renal failure is less predictable and may not be apparent for several weeks. Our data shows that normalization of LDH may take at least 10 weeks in some cases. Improvement of renal function by one stage may take one year. The slowness of renal function recovery has important clinical implications. Patients with advanced renal failure due to aHUS should not be rushed to kidney transplantation within one year (or even longer) of eculizumab treatment. On the other hand, if a patient has stable renal function and no thrombocytopenia, MAHA, or extrarenal complications, eculizumab therapy is unlikely to help confirm or refute the presumptive diagnosis of aHUS. For asymptomatic stable patients whose diagnosis of aHUS is not yet confirmed, it is prudent to withhold immediate eculizumab therapy and wait for the results of molecular testing. Nevertheless, the patients should be closely monitored for any change that would require immediate therapeutic intervention.

Case 5, who developed TMA after 7 courses of chemotherapy with gemcitabine and carboplatin, did not respond to eculizumab therapy. Before the onset of her renal dysfunction, her chemotherapy typically suppressed her platelet count to a nadir by days 7-8, recovering in time for her next scheduled chemotherapy at week 3. Therefore, the lack of increase in the platelet count following eculizumab therapy was not due to bone marrow suppression by her chemotherapy. Together with the lack of improvement in her mental status and renal function, it was very unlikely that the TMA of Case 5 was due to uncontrolled complement activation.

In summary, eculizumab therapy leads to rapid resolution of thrombocytopenia and extrarenal symptoms after the first dose. The resolution of MAHA and improvement of renal function is less predictable and may not occur for weeks to months. Concurrent plasma exchange impedes the response to eculizumab and should be avoided unless there is a compelling reason for continuation. Anticomplement therapy is ineffective for gemcitabine/carboplatin associated TMA.

## Figures and Tables

**Table 1:** Characteristics of the patients.

Case	Age, y	Gender	Comorbidity	Presenting features	Pathology	Molecular defects
1	61	F	None	Abdominal pain, diarrhea, and confusion	None available	None detected

2	57	F	3 months after auto-HCT for advanced multiple myeloma; MM in remission	Abdominal pain, diarrhea, vomiting, and confusion	None available	Anti-CFH, *CFHR1-CFHR3* +/−, and CFB c.1697A>C, p.Glu566Ala∗

3	50	M	None	Abdominal pain, nausea, vomiting, headache, gross hematuria, confusion, and hypertension (highest blood pressures 220/146 mmHg)	Kidney biopsy: TMA	None detected

4-1	22	F	Gestation at 22 weeks	Anorexia, anasarca, scotoma, and hypertension (highest blood pressures 205/134 mmHg)	Kidney biopsy: TMA	CFH c.3607C>T, p.Arg1203Trp∗∗
4-2			3 months after discontinuing eculizumab	Nausea, vomiting, dyspnea, and hypertension (highest blood pressures 221/159 mmHg)		

5	66	F	Gemcitabine/carboplatin and cholangiocarcinoma	Fatigue, somnolence, and confusion	Kidney biopsy: TMA	None detected

^*^An uncommon variant that is found in chimpanzee's CFB, which is 99% identical to human CFB.

∗∗A rare variant (prevalence of CT genotype, 0.001) in the CFH SCR-20 domain of uncertain consequence.

Abbreviations: CFH: complement factor H; HCT: hematopoietic stem cell therapy; MM: multiple myeloma; TMA: thrombotic microangiopathy.

**Table 2:** Laboratory findings at presentation.

Case	WBC	Hb	Platelet	LDH	BUN	Cr	Cr_max_	ADAMTS13	Plasma exchange
	×10^9^/L	g/L	×10^9^/L	U/L	mg/dL	mg/dL	mg/dL	%	Number of sessions
1	10.1	125	52	3,330	76	2.6	6.5 on day 3	86	17 for 19 days
2∗	12.5	85	99	1,271	39	3.37	—	55	15 for 18 days
3	10.48	95	96	1,471	55	4.84	5.99 on day 32	79	1 for 1 day
4-1	13.59	71	65	910	28	2.51	4.02 on day 17	85	6 for 6 days
4-2	5.07	84	63	770	46	5.52	6.86 on day 2	—	0
5∗∗	2.69	111	57	892	71	5.43	5.53 on day 2	74	5 for 6 days

^*^The patient was on hemodialysis at the time of transfer.

∗∗The patient received her last dose of gemcitabine and carboplatin 3 weeks before presentation.

Normal ADAMTS13 activity, >67%; Cr_max_: maximal serum creatinine.

**Table 3:** Responses to eculizumab therapy.

Case	Day	Hb	Platelet	LDH	Cr	Extrarenal complications	Days to normal values∗		Days to
		g/L	×10^9^/L	U/L	mg/dL		Platelet	LDH	CKD ↓ 1 stage
1	0	83	134	884	1.13	Intermittent confusion, and anasarca	≤7	>21	>21
	7	85	186	965	1.18	Resolved			

2	0	98	81	902	3.47	Somnolence, confusion, and vomiting	≤7	70	378, 483
	7	91	162	859	4.45	Resolved			

3	0	86	131	1,235	5.12	Headache, confusion, abdominal pain, nausea, and vomiting	≤7	14	63
	7	92	303	672	4.41	Resolved and HTN stabilized			

4-1	0	71	105	250	3.79	Anasarca, headache, and visual scotoma	3	14	14
	7	71	198	NA	2.66	Resolved			
4-2	0	84	63	770	5.52	Nausea, vomiting, and dyspnea	6	14	14
	6	83	230	321	NA	Resolved			

5	0	98	65	693	3.99	Somnolence and intermittent confusion	>84	>84	>84
	7	85	42	818	4.06	No improvement			

^*^Since the tests were not performed daily, the actual days may be less than the given values.

CKD: chronic kidney disease.

**Table 4:** Resolution of thrombocytopenia following initiation of eculizumab therapy for aHUS.

	Without concurrent plasma exchange		With concurrent plasma exchange	
Source	Steady increase by day 3	Normalized in ≤7 days	Steady increase by day 3	Normal in ≤7 days
Literature	10/10	10/12	1/5	0/5
This series	5/5	5/5	0	—

Total	15/15	15/17	1/5∗	0/5∗

Numerator indicates the number of cases with response; denominator is the number of cases with available data.

^*^ *P* = 0.001 and 0.0008, respectively, versus its counter group without plasma exchange (Fisher's exact test).
